# Does threat trigger prosociality? The relation between basic individual values, threat appraisals, and prosocial helping intentions during the COVID-19 pandemic

**DOI:** 10.1007/s12144-023-04829-1

**Published:** 2023-06-13

**Authors:** Emanuele Politi, Jasper Van Assche, Adrian Lüders, Sindhuja Sankaran, Joel Anderson, Eva G.T. Green

**Affiliations:** 1 Center for Social and Cultural Psychology, Faculty of Psychology & Educational Sciences, KU Leuven, Tiensestraat 102 B, 3000 Leuven, Belgium; 2 Laboratory of Social Psychology, Institute of Psychology, University of Lausanne, Lausanne, Switzerland; 3 Department of Developmental, Personality, and Social Psychology, Ghent University, Ghent, Belgium; 4 Center for Social and Cultural Psychology, Université Libre de Bruxelles, Bruxelles, Belgium; 5 Department of Communication Science, University of Hohenheim, Stuttgart, Germany; 6 Center for Social Cognitive Studies, Jagiellonian University Krakow, Kraków, Poland; 7 School of Behavioral and Health Sciences, Australian Catholic University, Queensland, Australia; 8 Psychology institute, Sai University, Channai, India

**Keywords:** COVID-19, Basic individual values, Threat appraisals, Prosociality, Helping

## Abstract

Prosociality is often considered as quintessential in coping with the threats of health emergencies. As previous research has suggested, prosocial behaviors are shaped by both dispositional factors and situational cues about the helping situation. In the present research, we investigated whether “bonding” types of prosociality, helping directed towards close others within one’s social network, and “bridging” types of prosociality, helping directed towards vulnerable people across group boundaries, are predicted by basic individual values and threat appraisals concerning COVID-19. During the pandemic, we conducted a cross-sectional study in the US and India (*N*_*total*_ = 954), using the Schwartz value inventory and a multifaceted measure of threat assessment to predict prosocial helping intentions. After controlling for other value and threat facets, self-transcendence values and threat for vulnerable groups uniquely predicted both bonding and bridging types of prosociality. Furthermore, threat for vulnerable groups partially mediated the effect of self-transcendence on prosocial helping intentions: People who endorsed self-transcendent values were particularly concerned by the effect of the pandemic on vulnerable groups, and thus willing to engage in prosocial behaviours to help those in need. Our findings support the idea that prosociality is stimulated by empathic concerns towards others in need and underline the importance for future research to consider the broad spectrum of threats appraised by people during health emergencies.

**Supplementary Information:**

The online version contains supplementary material available at 10.1007/s12144-023-04829-1.

## Introduction

The COVID-19 pandemic has profoundly affected individuals and societies worldwide. The public health emergency that began during the early months of 2020, and the stringent measures enforced to control it, constituted an unprecedented and prolonged threat for individuals worldwide (Mertens et al., 2022), with highly disruptive economic and social effects for all societies (ECDC, 2020). In the face of a challenge of such magnitude, researchers have underscored the importance of coping with stressors while promoting collective prosocial responses to overcome pandemic side effects for individuals and communities alike (e.g., Brooks et al., 2020; Jetten et al., 2020; Politi et al., 2022).

In the aftermath of significant life challenges, prosociality has been consistently associated with healing processes of post-traumatic growth (e.g., Tedeschi et al., 1998; Wlodarczyk et al., 2016). From a general threat-regulatory perspective, being prosocial in the face of a threat can be functional in that it permits individuals to overcome threat-induced inhibition and get back into threat-alleviating actions (Jonas et al., 2014; see also Vollhardt & Staub, 2011). One explanation for this effect is that people’s intrinsic values may serve as a guide for action when navigating psychological consequences of threat (Lüders et al., 2016). In other words, threats may trigger coping processes that resolve the threatening experiences together with people´s intrinsic values. Thus, in the context of the COVID-19 outbreak, identifying processes whereby values transform threat-induced inhibitions into prosocial helping behaviors may help promote individual recovery (Padilla-Walker et al., 2022; Ramkissoon, 2020, 2022). What is more, prosociality is not only associated with individual recovery, but it may favour the long-run recovery of communities and society (Compare, et al., 2021; Elcheroth & Drury, 2020; Muldoon et al., 2017; Rimé, 2020; Vignoles et al., 2021; Wlodarczyk et al., 2017). It is therefore essential to address the dispositional and contextual factors associated with prosocial responses to the COVID-19 epidemic, to collectively grow from this health emergency and be better prepared for future challenges.

Prior research has shown a link between previous natural disasters or disease outbreaks, and prosociality, both at the individual, group, and societal level (see Politi et al., 2022 for a review). By bridging research on interpersonal differences in prosocial values and general threat-regulation processes, in this paper we investigate how dispositional factors (i.e., basic individual values) relate to prosociality in the acute phases of the COVID-19 outbreak, via individual appraisals of situational threats triggered by the pandemic. In so doing, we show that relatively stable and trans-situational value orientations can trigger threat regulatory processes associated with emphatic concerns towards others in need. The integrated study of dispositional (values) and situational (threat appraisals) factors, we argue, will shed light on specific recovery strategies in the COVID-19 context and beyond.

### Basic individual values and prosociality

Prosociality can be defined as a “set of voluntary actions one may adopt to help, take care of, assist, or comfort others” (Caprara et al., 2005, p. 77; See also Politi et al., 2023). Such emphatic concerns are believed to be enduring attributes of the individual, rooted in dispositional factors like individual values (Caprara et al., 2012; Penner et al., 1995). According to Schwartz’s seminal theory on basic individual values (Schwartz, 1992; Schwartz et al., 2012), values can be considered trans-situational and cross-cultural life goals organized around a limited number of stable subdimensions: *self-enhancement* (i.e., emphasis on the pursuit of self-interests and relative success and dominance over others), *openness to change* (i.e., emphasis on independence of thought, action, and feelings and readiness for change), *conservation* (i.e., emphasis on order, self-restriction, preservation of the past, and resistance to change), and *self-transcendence* (i.e., emphasis on the welfare and interests of others). Self-transcendence is of particular relevance to the current study. Indeed, people high in *self-transcendence* values exhibit a willingness to sacrifice their self-interest in favour of others and cooperate during group tasks, features that constitute the psychological foundations of prosociality (Caprara & Steca, 2007; Schwartz, 2010).

In the specific context of the COVID-19 outbreak, Politi et al. (2021) confirmed the specific relationship between self-transcendence values and prosociality. That is, compared to other values orientations, ideological beliefs, and political values, people endorsing self-transcendence values reported higher prosocial intentions and behaviours alike (see also Russo et al., 2022; Wolf et al., 2020). This was true for both “bonding” types of prosociality, namely acts of helping directed towards close others within one’s own social network, such as friends and neighbours; as well for “bridging” types of prosociality, namely acts of helping directed towards vulnerable people across group boundaries, such as homeless people and refugees populations (Politi et al., 2022; see also Zagefka, 2021, for a similar distinction between ingroup and outgroup helping).

The present research thus seeks to expand the above findings by focusing on whether different situational threat experiences in the context of the COVID-19 outbreak mediate the well-established link between self-transcendence and prosociality. Furthermore, the research examines whether these processes are limited to bonding types or also apply to bridging types of prosociality. Accordingly, we provide empirical evidence of threat regulatory processes that guide prosocial values into prosocial responses towards others in need.

### Threat appraisals during the COVID-19 outbreak

Disasters and pandemic diseases have frequently been associated with threat perception and regulation (Jonas et al., 2014). Accordingly, Brooks et al. (2020) provided an early overview of the most prevalent stressors triggered by past infectious diseases. They contested that the pandemic had a psychological impact on the population solely due to its direct health ramifications, emphasizing instead a larger array of secondary threats exacerbated by disease outbreaks (see also Coelho et al., 2020, for a distinction between primary and secondary effects of the current COVID-19 pandemic). Along similar lines, Politi et al. (2022) systematically reviewed prior knowledge on disease outbreaks and natural disasters, expanding the definition of threat. They suggested an intra-psychological regulatory process to include all external or internal stressors appraised as a potential danger to the personal and social self, communities, and entire societies. Building on these concepts, Anderson et al. (2021) recently developed a comprehensive analytical approach, finding evidence for ten distinct threat facets in relation to the COVID-19 outbreak.

The above mentioned ten threat facets were also adopted in the present research: (1) *Life threat* refers to the personal fear of getting the virus and eventually dying from health complications (e.g., Ahorsu et al., 2020). (2) *Existential threat* refers to psychological needs frustrations, such as loss of meaning, control, and directionality (e.g., Arpaci et al., 2020). (3) *Relational threat* refers to loneliness and isolation due to the disruption of social ties during the confinement (e.g., Kira et al., 2021). (4) *Lifestyle threat* refers to widespread concerns for the disruption of daily routines and planned activities (e.g., Mertens et al., 2021). (5) *Financial threat* refers to fears of job loss and financial struggles (e.g., Rogers, et al., 2021). (6) *Supply threat* refers to worries and anxieties due to shortages of essential products, such as food and medicines (e.g., Arpaci et al., 2020). (7) *Healthcare threat* refers to the sense of danger for the imminent collapse of the healthcare system (e.g., Mertens et al., 2021). (8) *Social fabric threat* refers to perceived disruption of cultural values and social order (e.g., Kachanoff et al., 2020). (9) *Political dysregulation threat* refers to worries for authoritarian surveillance and coercion of individual freedoms (e.g., Oleksy et al., 2021). (10) *Threat for vulnerable groups*, finally, refers to altruistic concerns for those who were most suffering the negative impacts of the pandemic (e.g., Sloan et al., 2021).

This research disentangles different dimensions of threat appraisal, linking them to both individual values as predictors and prosociality as a regulatory response. The identification of specific threat regulatory processes mediating the relationship between basic individual values and prosociality enhances our understanding of the psychological mechanisms that promote growth-oriented responses to threat. Recently, some empirical findings suggest that differences in individual values trigger different pandemic-implied threat appraisals (Lemay et al., 2021; Padilla-Walker et al., 2022), some of which are related to prosocial responses, while others are related to antisocial responses (Kruglanski et al., 2021; Serrano-Montilla et al., 2021; Tse et al., 2021; Vieira et al., 2020). Assuming that prosociality is most often the result of empathic concerns for others in need (e.g., Batson & Powell, 2003; Graziano et al., 2007; Yue & Yang, 2022), we expect that people moved by self-transcendent values appraise the pandemic as a potential threat for others in need (i.e., the tenth threat facet identified by Anderson et al., 2021), rather than merely focusing on their personal concerns. This ‘other-oriented’ mindset as a response to the COVID-19 outbreak should be associated to both bonding and particularly bridging prosociality.

### The Present Study

While exploring the broad correlational relations between basic individual values and threat appraisals, the present study tests whether threat for vulnerable groups mediates the link between self-transcendent values and both bonding and bridging types of prosociality. We test these associations with both forms of prosociality, to provide exploratory evidence if specific threat regulatory processes related to dispositional differences generalize to helping intentions directed towards outgroup members. Specifically, we formulated the following hypotheses:

H1: Self-transcendence values should be positively related to prosocial helping intentions.

H2: Threat for vulnerable groups should be positively related to prosocial helping intentions.

H3: Threat for vulnerable groups should mediate the effect of self-transcendence values on prosocial helping intentions such that:

a. The more people endorse self-transcendence values, the more they should experience a threat for vulnerable groups.

b. The more people experience a threat for vulnerable groups the more they should be willing to engage in prosocial helping actions.

We test these hypotheses based on data collected in the US and India in September 2020. At the time of data collection, more than one-third of the total COVID-19 cases detected worldwide came from these two countries, making this research particularly timely the context of the COVID-19 pandemic. Not only do the US and India differ in terms of social, political, and economic demographics, but they also diverge substantially in terms of shared ideological worldviews and cultural patterns of individualism and collectivism (Elischberger et al., 2018; see also Verma & Triandis, 1999). By estimating cross-cultural invariances in the way threats are appraised and associated with basic individual values, on the one hand, and prosocial responses to the COVID-19 outbreak, on the other hand, the multi-group analyses conducted on the data obtained allow us to generalize threat regulatory processes beyond frequently studied Western, Educated, Industrialized, Rich, and Democratic countries (Henrich et al., 2010; Muthukrishna et al., 2020).

## Method

### Participants

Nine-hundred and fifty-four participants in total (age range: 18–76 years, *M* = 33.73, *SD* = 11.89; Gender: male = 460, female = 469, gender diverse = 16) were recruited from both the US and India. For the US sample (*n* = 471), all participants were recruited via the online platform Prolific and were paid at the rate of five USD per hour. For the Indian sample (*n* = 473), 164 participants were recruited through Amazon’s Mechanical Turk and were reimbursed one USD in exchange for their participation, while 309 participants were recruited via snowballing sampling. Socio-demographics for each subsample are reported in Table 1. In terms of country differences, the age range did not differ between the two countries, *F* (942,1) = 0.142, *p* = .71. There were slightly more female participants in the US sample and more male participants in the Indian sample, χ^2^(1, *N =* 929) = 7.06, *p* = .008. Employment status differed between the US and India, χ^2^(5, *N =* 910) = 40.94, *p* < .001, with more full-time workers coming from India, and more part-time and unemployed workers coming from the US. Among those participants who were active in the labour market at the time of the COVID-19 pandemic, more workers coming from India were able to keep their job as compared to US workers, χ^2^(2, *N =* 617) = 21.58, *p* < .001. Of those who were working, no differences in the working environment were found between the two countries, χ^2^(2, *N =* 580) = 1.69, *p* = .430. Finally, more participants in India reported having already contracted COVID-19 than the US, χ^2^(1, *N =* 945) = 41.02, *p* < .001.

**Table 1:** Socio-demographic characteristics of participants in the US and India

	Country				
	US		India		
Gender	*n*	*%*	*n*	*%*	
Male	213	45.1%	256	54.1%	
Female	249	52.8%	211	44.6%	
Diverse	10	2.1%	6	1.2%	
Mean Age (SD)	32.23 (11.92)		35.23 (11.68)		
Employment status					
Full-time	196	41.5%	266	56.2%	
Part-time	81	17.2%	51	10.8	
Other capacity	9	1.9%	14	3.0%	
Unemployed	71	15%	26	5.5%	
Retired	14	3.0%	21	4.4%	
Student	83	17.6%	78	16.5%	
Other	18	3.8%	17	3.6%	
Keep job					
Yes	34	7.2%	48	10.2%	
Not sure	229	48.5%	264	55.8%	
No	23	4.9%	19	4.0%	
Not applicable or missing	203	39.4%	161	30.0%	
Workplace					
Home	162	34.3%	203	42.9%	
Usual workplace	98	20.8%	103	21.8%	
Other location	8	1.7%	6	1.3%	
Not applicable or missing	204	43.2%	180	34.0%	
COVID-19 infected					
Yes	110	23.3%	203	42.9%	
No	362	76.7%	270	57.1%	

### Measures

For each measure separately, we conducted a series of Multi-Group Confirmatory Factor Analyses (CFA) with robust Satorra-Bentler standard errors to correct for multivariate normality using the R package “Lavaan” (Rosseel, 2012). Cut-off criteria of fit measures were derived from Hu and Bentler (1999). Differences between models were assessed using changes in the Chi-square / degrees of freedom ratio, the Bayesian information criterion (BIC) and the Comparative fit index (CFI), as suggested by Vandenberg and Lance (2000). Here, we report the final constrained measurement models. To get the exact details for each indicator and full information about measurement and structural group invariance between India and the US, please see the Supplementary Online Materials (SOM).

**Basic individual values.** We assessed Basic individual values with 16 items derived from the short version of the Portrait Values Questionnaire (PVQ-21) developed by Schwartz et al. (2012). For each portrait, respondents indicated how similar the described person was to themselves on a scale ranging from 1 (*Not at all like me)* to 6 (*Very much like me*). Participants’ self-reported gender was matched with the portraits. Five items measured our focal independent variable, namely *self-transcendence* values (e.g., “He/she thinks it is important that every person in the world be treated equally. He/she wants justice for everybody, even for people he/she doesn’t know”); four items measured values related to *openness to change* (e.g., “Thinking up new ideas and being creative is important to him/her. He/she likes to do things in his/her own original way”); four items measured values related to *self-enhancement* (e.g., “Being very successful is important to him/her. He/she likes to impress other people”); three items measured values related to *conservation* (e.g., “He/she believes that people should do what they’re told. He/she thinks people should follow rules at all times, even when no-one is watching”). Model fit of the measure was good: *χ*^*2*^(209) = 444.17, *p* < .001; CFI = 0.93; RMSA = 0.05, 90% CI [0.04; 0.05], *p* = .62; SRMR = 0.06 (see Table S1 in the SOM for the exact wording, latent factors, and measurement invariance of the PVQ-21).

**COVID-19 multifaceted threats.** We assessed 10 threat subdimensions related to the COVID-19 outbreak using the 30-item COVID-19 Multifaceted Threat Scale previously developed by Anderson et al. (2021). For each statement, respondents indicated to what extent they felt worried or concerned about the pandemic situation on a scale ranging from 1 (*Not at all concerned*) to 7 (*Extremely concerned*). Three indicators measured our central mediator, namely *threat for vulnerable groups* (e.g., “Homeless people are not able to protect themselves”). Three indicators measured each of the remaining threat facets: *personal health* (e.g., “I might catch COVID-19”); *existential* (e.g., “My life has less meaning these days”); *relational* (e.g., “I miss my friends”); *lifestyle* (e.g., “I don’t know when my next vacation will be”); *basic supplies* (e.g., “There could be food shortages in the supermarkets”); *financial* (e.g., “I might run of money”); *healthcare system* (e.g., “Medical staff are unable to keep up with what is needed of them”); *social fabric* (e.g., “There are too many irresponsible people who do not respect social distancing”); *political* (e.g., “The government’s response to the coronavirus is being used for political gains”). Model fit was excellent: *χ*^*2*^(757) = 1170.98, *p* < .001; CFI = 0.97; RMSA = 0.03, 90% CI [0.03; 0.04], *p* > .99; SRMR = 0.05 (see Table S2 in the SOM for the exact wording, latent factors, and measurement invariance of the COVID-19 Multifaceted Threat Scale).

**Bonding and bridging prosocial helping intentions.** Bonding and bridging types of prosociality were measured using items adapted from Politi et al. (2021).Three indicators measured *bonding prosocial helping intentions* directed towards people physically and psychologically close to participants (e.g., “I am willing to do grocery shopping for those people in my neighbourhood who are in need”). Three indicators measured *bridging prosocial helping intentions* directed towards people physically and psychologically distant to participants (e.g., “I am willing to sign a petition to ask for international solidarity towards other countries that are having hard times in dealing with the corona crisis”). Model fit was excellent, *χ*^*2*^(26) = 52.15, *p* = .002; CFI = 0.99; RMSA = 0.05, 90% CI [0.03; 0.06], *p* = .66; SRMR = 0.05 (see Table S3 in the SOM for the exact wording, latent factors, and measurement invariance of the scale used to measure bonding and bridging prosocial intentions).

## Results

### Descriptive statistics and covariations

At first, to explore covariations between basic individual values, COVID-19 multifaceted threats, and bonding and bridging prosocial intentions, the three scales were included in a unique Multi-group CFA. Model fit was excellent: *χ*^*2*^(2449) = 3592.28, *p* < .001; CFI = 0.95; RMSA = 0.03, 90% CI [0.03; 0.04], *p* > .99; SRMR = 0.06. Meaningful associations between latent factors in India and the US are summarized below. Readers are referred to the SOM to check for country invariance (Table S4) in the latent variance/covariance matrix (Table S5).

As for basic individual values and prosocial intentions, self-transcendence and openness to change showed positive associations with both bonding and bridging prosocial helping intentions in both the US and India. These findings were in line with previous evidence on the positive links of self-transcendence and openness to change with prosociality (Politi et al., 2021). Interestingly, conservation showed positive associations with bonding and bridging prosocial helping intentions in India but not in the US. Finally, self-enhancement on the other was unrelated to both bonding and bridging prosocial intentions in the US and in India.

As for basic individual values and COVID-19 threat appraisals, self-transcendence showed positive associations with health, relational, supply, healthcare system, social fabric, political, and vulnerable group threat appraisals, in both the US and India. These findings suggest that self-transcendence values activate mainly other-oriented contextual threat clues, such as concerns for significant others and society more broadly (for a similar finding, see Lemay et al., 2021). In both countries, self-enhancement was positively associated with health, existential, relational, lifestyle, and financial threat. These findings suggest that self-enhancement values activated mainly self-oriented contextual threat clues, such as loss of meaning, continuity in one’s lifestyle, and financial security (Kruglanski et al., 2021). Concerning the values related to openness to change, however, the two countries differed. In the US, openness to change was positively associated with relational, lifestyle, supply, and financial threat. In India, openness to change was instead positively associated with the healthcare system, social fabric, and vulnerable group threats. Again, concerning the values related to conservation, the two countries differed. In the US, conservation was positively associated with relational, lifestyle, and supply threats, as well as negatively associated with healthcare system, social fabric, political, and vulnerable group threats. In India, conservation was instead positively associated with health, relational, supply, and financial threats, but also with healthcare system, social fabric, and vulnerable group threats.

As for COVID-19 threat appraisal and prosociality, in line with our hypotheses, threat for vulnerable groups showed positive associations with bonding and bridging prosocial helping intentions. Bridging prosocial helping intentions also showed positive associations with health, relational, supply, healthcare system, social fabric, and political threats. As for bonding prosocial helping intentions, in India (but not in the US) they showed positive associations with health, healthcare system, social fabric, and political threats. In line with previous findings, these results suggest that prosociality is related to a psychological sense of community and collective self-construal (Compare et al., 2021; Padilla-Walker et al., 2022).

### Hypothesis testing

**Associations between self-transcendence values and prosocial helping intentions (H1).** Self-transcendence, openness to change, and conservation (i.e., the three higher-ordered factors that showed positive associations with either bridging or bonding prosocial helping intentions) were included as endogenous factors in a Multi-group Structural Equation Modelling (SEM), and bonding and bridging prosocial intentions were modelled as endogenous factors. Latent factors were allowed to covary. Age, gender, infection history, social distance practices, and subjective social-economic status were used as covariates (see Table 2). Model fit was excellent: *χ*^*2*^(526) = 1035.89, *p* < .001; CFI = 0.91; RMSA = 0.05, 90% CI [0.04; 0.05], *p* = .96; SRMR = 0.06. Results were in line with H1 and showed that self-transcendence uniquely predicted both bonding, *b* = 0.85 (0.13), *p* < .001, and bridging prosocial helping intentions, *b* = 0.98 (0.13), *p* < .001. No differences in slopes were observed between India and the US (for all other estimates, *p* > .05). In line with previous findings, when we controlled for other basic individual values, the only value significantly associated with prosociality was self-transcendence (Politi et al., 2021).

**Table 2:** Structural model estimating the effects of basic individual values on bonding and bridging prosocial helping intentions

*Threats*	Bonding prosocial helping intentions			Bridging prosocial helping intentions		
	*b*	*SE*	*p*	*b*	*SE*	*p*
Self-transcendence	0.85	0.13	< 0.001	0.98	0.13	< 0.001
Openness to change	0.18	0.09	0.05	0.12	0.09	0.21
Conservation	–0.02	0.07	0.81	*–*0.13	0.08	0.09
*Control variables*						
Gender	0.22	0.09	0.01	0.08	0.10	0.39
Age	0.007	0.004	0.07	*–*0.02	0.004	< 0.001
Infection history	*–* 0.23	0.09	0.02	*–* 0.17	0.10	0.10
Social distancing practices	0.05	0.04	0.17	0.06	0.04	0.15
Subjective SES	0.15	0.04	< 0.001	0.17	0.04	< 0.001

*Note*: Estimates extracted from multi-group SEM using the R package “Lavaan”. Country (the US vs. India) was used as grouping variable. Self-enhancement was excluded because showed no significant covariations with neither bonding nor bridging prosocial intentions. Statistical controls were coded as follow: Concerning gender, female participants (1) were contrasted against male (0) participants, gender diverse was excluded due to limited frequency. Concerning infection history, participants who had not experienced COVID-19 (1) were contrasted against participants who experienced it (0). Concerning social distancing practices, the variable asked to what extent participants were able to respect social distancing in their daily activities, on a 7-point scale from not at all (1) to extremely well (7). Concerning subjective SES, the variable asked whether participants considered themselves as far worse off than others (1) or far better off (7) than other people in their country. Model fit: *χ*^*2*^ (526) = 1035.89, *p* < .001; CFI = 0.91; RMSA = 0.05, 90% CI [0.04; 0.05], *p* = .96; SRMR = 0.06

**Associations between threat for vulnerable groups and prosocial intentions (H2).** Since all COVID-19 threat appraisals showed positive associations with prosociality, they were all modeled as exogenous factors. Bonding and bridging prosocial helping intentions, in turn, were modeled as endogenous factors. Latent factors were allowed to covary (see Table 3). Model fit was satisfactory: *χ*^*2*^(1569) = 3010.48, *p* < .001; CFI = 0.92; RMSA = 0.05, 90% CI [0.04; 0.05], *p* > .99; SRMR = 0.08. Results were in line with H2 and showed that threat for vulnerable groups uniquely predicted both bonding, *b* = 0.42 (0.08), *p* < .001, and bridging prosocial helping intentions, *b* = 0.59 (0.08), *p* < .001. No differences in slopes were observed between India and the US. Although not hypothesized, residual effects of relational threat were found both in India and the US on bonding, *b* = 0.13 (0.06), *p* = .05, and bridging prosocial helping intentions, *b* = 0.16 (0.07), *p* = .01. Conversely, existential threat was related to reduced bonding prosocial helping intentions, *b* = –0.14 (0.05), *p* = .004, but unrelated to bridging prosocial helping intentions, *b* = –0.07 (0.05), *p* = .15. Curiously, healthcare system threat reduced bonding prosocial helping intentions in the US, *b* = –0.23 (0.08), *p* = .005, but not in India *b* = 0.11 (0.09), *p* = .21. All other *p*s > 0.13.

**Table 3:** Structural model estimating the effects of multidimensional COVID-19 threats on bonding and bridging prosocial helping intentions

*Threats*	Bonding prosocial helping intentions			Bridging prosocial helping intentions		
	*b*	*SE*	*p*	*b*	*SE*	*p*
Vulnerable group	0.42	0.08	< 0.001	0.59	0.08	< 0.001
Political	–0.06	0.10	0.53	–0.09	0.11	0.38
Social fabric	–0.02	0.08	0.82	0.02	0.08	0.85
Healthcare system	0.11 *–0.23*	0.09 .*08*	0.21 *0.005*	–0.07	0.08	0.40
Financial	0.07	0.04	0.06	0.03	0.03	0.32
Basic supply	*–* 0.02	0.04	0.69	0.03	0.05	0.57
Lifestyle	*–*0.05	0.04	0.24	–0.06	0.04	0.13
Relational	0.13	0.06	0.05	0.16	0.07	0.01
Existential	*–*0.14	0.05	0.004	*–*0.07	0.05	0.15
Personal health	0.001	0.08	0.99	*–*0.02	0.08	0.80
*Control variables*						
Gender	0.28	0.09	0.002	0.05	0.10	0.63
Age	0.004	0.004	0.35	*–*0.02	0.004	< 0.001
Infection history	*–* 0.27	0.10	0.006	*–* 0.17	0.10	0.10
Social distancing practices	0.04	0.04	0.26	0.03	0.04	0.38
Subjective SES	0.15	0.04	< 0.001	0.17	0.04	< 0.001

*Note*: Results extracted from multi-group SEM using the R package “Lavaan”. Country (the US vs. India) was used as grouping variable. When slopes differed between countries, US estimates are reported in italic. Statistical controls were coded as follow: Concerning gender, female participants (1) were contrasted against male (0) participants, gender diverse was excluded due to limited frequency. Concerning infection history, participants who had not experienced COVID-19 (1) were contrasted against participants who experienced it (0). Concerning social distancing practices, the variable asked to what extent participants were able to respect social distancing in their daily activities, on a 7-point scale from not at all (1) to extremely well (7). Concerning subjective SES, the variable asked whether participants considered themselves as far worse off than others (1) or far better off (7) than other people in their country. Model fit: *χ*^*2*^ (1569) = 3010.48, *p* < .001; CFI = 0.92; RMSA = 0.05, 90% CI [0.04; 0.05], *p* > .99; SRMR = 0.08

**Fig. 1:**
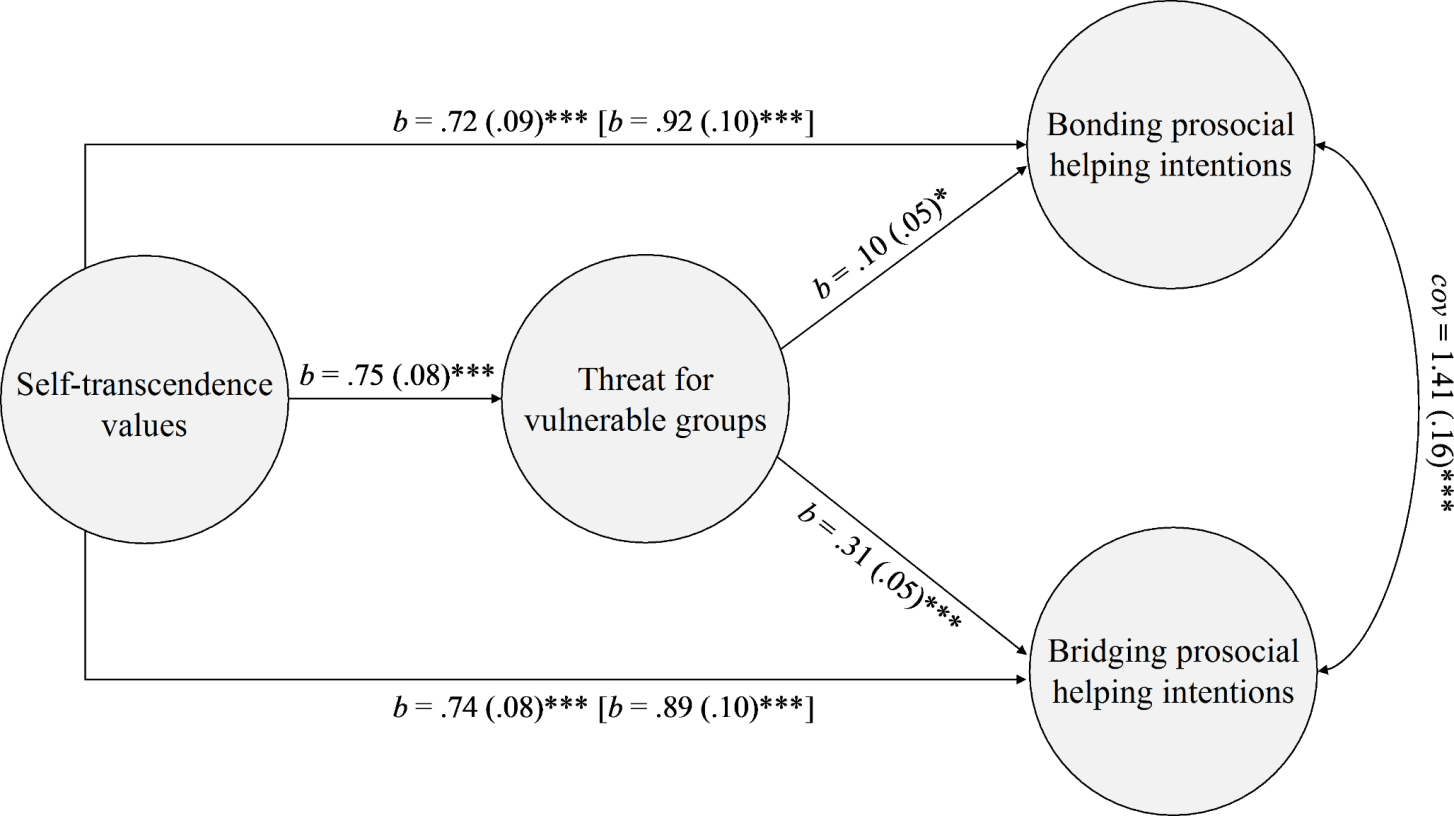
*Note:* Direct and indirect effects of self-transcendence on bonding and bridging prosocial helping intentions via threat for vulnerable groups. (*Note*: Estimates extracted from multi-group SEM using the R package “Lavaan”. Total effects of self-transcendence values on bonding and bridging prosocial helping intentions are reported in square brackets. Indirect effects were, *b* = 0.07 (0.03), *p* = .03, and, *b* = 0.23 (0.04), *p* < .001, on bonding and bridging prosocial helping intentions respectively. Country (the US vs. India) was used as grouping variable. Gender, age, infection history, social distancing practices, and subjective SES were used as statistical controls. Model fit: *χ*^*2*^(316) = 577.43, *p* < .001; CFI = 0.95; RMSA = 0.04, 90% CI [0.04; 0.05], *p* = .99; SRMR = 0.06. *** *p* < .001, * *p* < .05)

**Indirect effect of self-transcendence on prosocial helping intentions via threat for vulnerable groups (H3).** As a final step, we modelled self-transcendence as exogenous independent variable, threat for vulnerable groups as endogenous mediator, and bonding and bridging prosocial helping intentions as endogenous dependent variables (see Fig. 1). Model fit was satisfactory: *χ*^*2*^(316) = 577.43, *p* < .001; CFI = 0.95; RMSA = 0.04, 90% CI [0.04; 0.05], *p* = .99; SRMR = 0.06. Results were in line with H3. Indeed, self-transcendence predicted threat for vulnerable groups, *b* = 0.75 (0.08), *p* < .001 (H3a). When controlling for self-transcendence, in turn, threat for vulnerable groups predicted both bonding, *b* = 0.10 (0.05), *p* = .05 and to a greater extent bridging prosocial helping intentions, *b* = 0.31 (0.05), *p* < .001 (H3b). A test for indirect effects confirmed that threat for vulnerable groups served as intermediary variable in the relationships between self-transcendence and both dimensions of prosocial helping intentions, i.e. bonding, *b* = 0.07 (0.03), *p* = .03, and bridging, *b* = 0.23 (0.04), *p* < .001. Importantly, the indirect effect was stronger for bridging than bonding prosocial helping intentions, Δ*χ*^*2*^(1) = 9.82, *p* = .01, ΔBIC = 13, ΔCFI = – 0.003. Neither of the two indirect effects significantly differed between India and the US. When threat for vulnerable groups was controlled for, the effect of self-transcendence remained significant, on both bonding, *b* = 0.73 (0.09), *p* < .001, and bridging prosocial helping intentions, *b* = 0.75 (0.08), *p <* .001. Yet, as compared to the total effect, the direct effect of self-transcendence on bonding and bridging prosociality helping intentions was smaller in magnitude, Δ*χ*^*2*^(2) = 6.74, *p* = .03, ΔBIC = 8, ΔCFI = – 0.001, thus suggesting that a significant part of the effect was mediated by threat for vulnerable groups.

## Discussion

A large number of studies conducted during the COVID-19 outbreak have outlined the importance of individual predispositions and worldviews in regulating people’s responses to pandemic challenges (e.g., Bonetto et al., 2021; Kruglanski et al., 2021; Tu et al., 2021; Wolf et al., 2020). Increasing attention has been dedicated to prosociality and its predictors, conceived as a collective remedy to protect individual wellbeing, safeguard communities, and recover societies from the negative impacts of the pandemic (Jordan et al., 2021; Ramkissoon, 2022; Zagefka, 2022). The present research not only corroborates previously established links between basic individual values and prosociality (e.g., Politi, et al., 2021; Schwartz et al., 2010), but also offers an innovative understanding of the underlying threat-regulatory processes involved (e.g., Reiss et al., 2020). Accordingly, we combined dispositional explanations of prosociality based on enduring and trans-situational individual characteristics, with situational threat-and-defence regulative processes, to predict prosocial responses. By comparing a variety of threats related to both the vulnerable self as well as to vulnerable others, we found evidence that the way in which individuals experience the pandemic as threatening (a) is shaped by their embraced values and (b) induces specific threat-regulatory responses.

Drawing on two large samples collected in the US and India in the acute phase of the pandemic (September 2020), we showed that other-oriented values such as self-transcendence activate empathic concerns (Galang et al., 2021; Pfattheicher et al., 2020; Varma et al., 2022), and due to this promote prosociality as a means of coping. Indeed, when controlling for all other value structures and threat facets, threats for vulnerable groups mediated the positive relationship between self-transcendence values and prosocial helping intentions. This pattern of results was robust and consistent across both the US and India. Furthermore, this indirect relationship was particularly accentuated for bridging types of prosocial helping intentions, wherein threats for vulnerable groups were more strongly related to helping distant others (e.g., donating to an organisation) than close others (e.g., helping a neighbours). In line with previous findings, empathic concerns for others in need seem particularly effective in bridging group cleavages and promoting intergroup helping (e.g., Graziano et al., 2007; Van Leeuwen & Zagefka, 2017; see also Zagefka, 2022).

While not predicted and mostly exploratory, results showed distinctive differences between India and the US concerning conservation values and their association with prosociality. In India (but not in the US), conservation values were positively related to bonding and bridging types of prosociality, implying that values related to self-restriction, order, security, and tradition hold a particular meaning in Indian society. It is known that the moral and social qualities valued in society have their roots in religion, philosophy and tradition (Krishnan & Manoj, 2008). Thus, from the perspective of Indian philosophy, one of the most important values that people are taught is that of ‘daanam’ or giving. This value is easily translated to the Western notion of prosocial behaviour. But according to Krishnan (2005) ‘daanam’ extends beyond prosociality and is also rooted in Indian traditional values. A perusal of Hindu scriptures reveals that ‘daanam’ is mentioned in several texts in a religious or spiritual background, and is one of many actions prescribed for attaining the highest goal in life, namely, spiritual salvation. Traditional Indian thought in fact upholds ‘daanam’ as something noble (Krishnan & Manoj, 2008).

Connecting it with values in contemporary psychology, social interdependence is a strong norm amongst Indians, strengthening the importance of helping people, both close family members and those outside the family circle. Furthermore, according to Hinduism, the majority religion practiced in India, there is a significant emphasis on the concept of reincarnation and Karma (action). Karma is the heart of Hinduism that implies that every ‘good’ action leads to ‘good’ outcomes. One such ‘good’ action is that of helping others. With better ‘Karma’ in one’s active life, the chances of being reincarnated in the highest form (human being) are higher. Similarly, Dharma is also an essential concept in Hinduism, which means duty’, ‘virtue,‘ and ‘morality.‘ Dharma is known as the power that maintains society and makes us morally conscientious allowing humans to act virtuously (Flood, 2009). Traditional Indians imbibe these ancient norms that guide them in everyday living (Mahalingam, 2007; Sankaran et al., 2017). This probably explains why conservation values were strongly and positively related to prosociality in the current Indian subsample.

Apart from these strengths and contributions, some limitations in our work must be acknowledged and overcome via future investigations. First, the correlational nature of our data impedes conclusive causal inferences. Basic individual values (i.e., our exogenous variable) are conceived as dispositional and trans-situational. Conversely, threat appraisals and solidarity intentions (i.e., our endogenous variables) are conceived as situational and incidental responses to the COVID-19 outbreak. Challenging the understanding of basic individual values as stable dispositions, however, some authors argued that unexpected and disruptive life changes can boost certain value orientations while attenuating others (Bardi et al., 2009). Others observed changes in values and worldviews after engaging in prosocial actions (Schlitz et al., 2010). More conclusive experimental evidence is needed to test the causal mechanisms that from basic individual values flow into threat appraisal and lead to prosociality. By manipulating the situational appraisal of threat, for instance, future investigations may discern whether self-oriented and other-oriented threats produce changes in prosociality and value orientations.

Another limit concerns the cross-cultural invariance of our measures, specifically but not limited to basic individual values. According to the seminal theory of Schwartz et al. (2001), basic individual values should be considered universal and cross-cultural. As explained in detail in the SOM, however, configural invariance was only partially met concerning basic individual values, implying slightly different value configurations in the two countries. By testing partial invariance, identifying and eventually removing problematic indicators, we avoided problems of inequivalences of the model forms and produced latent factors that were reliable in both countries. Value differences in the two countries may be a result of the severity of the pandemic in the two countries, especially because September 2020 was one of the worst months for India in terms of contagion and deaths (TNN, 2020). Recent studies have indeed indicated the malleable nature of values in the context of COVID-19 pandemic (Bojanowska et al., 2021; Bonetto et al., 2021). Accordingly, for all our variables, no scalar (strong factorial) invariance could be met, meaning that intercept means differed between the two samples. Although our first interest lied in testing covariances rather than mean differences between the latent constructs, this lack of scalar invariance may further signal different experiences of the pandemic in the US and India during the acute phases of the COVID-19 outbreak. Similarly, cross-cultural studies have gathered evidence that the pandemic experience differed depending on cultural and structural specificities, further exacerbated by different impacts of the pandemic on individuals, societies, and economies (e.g., Dean et al., 2021; Padilla-Walker et al., 2022; Pagliaro et al., 2021).

A general limitation to the growing amount of research produced during the pandemic’s emergency phase is that measurements and underlying assumptions may quickly become outdated. Threats that were appraised as acute stressors during the initial waves of virus insurgence may not have held in the following phases of virus containment and suppression. Conversely, other threats that were absent at the beginning rose in importance in the subsequent phases of the pandemic. As the pandemic has left important and lasting marks on world economies and societies, we believe that future research should move forward from an immediate emergency phase of the pandemic to a more far-looking programmatic stage of global resilience. More evidence is needed, for instance, to understand which personal predispositions and threat cues propel individuals in (i) adhering to or contesting vaccination campaigns, (ii) continuing or retreating from health protocols over the years, (iii) complying or opposing appeals for responsible behaviours, and, more generally, (iv) trusting or not trusting institutions and science to lead us out of emergencies collectively (Taylor et al., 2020; Turhan et al., 2022). By tackling the specific mechanisms linking individual values, threat appraisals, and prosocial responses in the acute phase of the pandemic, societies will be in a slightly more strategic position to anticipate future emergencies and design better recovery plans in eventual virus resurgence or other global challenges facing us.

## Conclusion

The COVID-19 pandemic in many ways posed an unprecedented threat to global humanity. In times of raising tensions between global powers, mass displacement due to torn conflicts, and a looming climate crisis, understanding how people respond to large-scale threats is an important transdisciplinary challenge. The present data stressed the need to consider threat as a multifaceted experience. Depending partially on their embraced basic values, individuals may experience and prioritize different crisis-related aspects as the most pressing and therefore engage in different coping responses. The findings of this paper underline the fact that subjective threat realities may differ even when objectively we may all be “sitting in the same boat.” Considering this fact is an important first step to help prevent conflictual social dynamics that can hamper collective threat management. In order to support sustainable large-scale threat management, future research should seek strategies to enhance prosocial coping responses also among those for whom such responses may not come naturally.

## Electronic supplementary material

Below is the link to the electronic supplementary material.

Supplementary Material 1

## Data Availability

Data and syntaxes are stored and freely available on the Open Science Framework via the following anonymous link: https://osf.io/pm2tf/?view_only=1a423af43a7a47f9a4825adca09a5622.
