# Supplementary material for: Does threat trigger prosociality? The relation between basic individual values, threat appraisals, and prosocial helping intentions during the COVID-19 pandemic
Source: Curr Psychol. 2023 Jun 13:1–13. Online ahead of print. doi: 10.1007/s12144-023-04829-1 (PMC10262135; doi:10.1007/s12144-023-04829-1)
Supplement: Supplementary file 1 — Supplementary Material 1 [file 12144_2023_4829_MOESM1_ESM.docx]

**Calculation of Measurement and Structural Group Invariance**

A series of Multi-Group Confirmatory Factor Analyses (CFA) using the R package “Lavaan” was first performed on Basic individual values, Multidimensional COVID-19 threats, and Prosociality separately, and then performed on all scales altogether (Rosseel, 2012) for both Indian and American samples. Cut-off criteria of fit measures were set as CFI ≥ .95, RMSEA ≤ .06, and SRMR ≤ .08 (Hu & Bentler, 1999). Differences between models were assessed using Chi-square statistics, changes (Δ) in Bayesian Information Criterion (BIC) and Comparative Fit Index (CFI), as suggested by Vandenberg (2002). When not indicated otherwise, slopes did not differ between countries.

As a first step, all parameters were allowed to vary between the US and India. As soon as configural invariance was met in both countries, we proceeded testing increasing levels of measurement invariance (Beaujean, 2014). Factor loadings were constrained one by one (weak metric invariance), followed by observed intercepts (strong scalar invariance), and indicator error terms (strict residual invariance). By constraining degrees of freedoms gradually, we held the advantage to measure increasing levels of partial invariance and to locate eventual group discrepancies rigorously.

As soon as measurement invariance was met, we included all measures in a unique CFA model and applied a similar procedure to test increasing levels of structural invariance. Latent variances were constrained one by one, followed by latent covariances.

**Measurement Invariance**

**Basic individual values.** We assessed Basic individual Values with 21 items derived from Schwartz and colleagues (2012). For each portrait, respondents indicated how similar the person was to themselves on a scale ranging from 1 (*Not at all like me)* to 6 (*Very much like me*). The 21 items replicated the multidimensionality proposed by Schwartz and colleagues (Schwartz et al., 2012). Originally, five indicators measured the 5 values covering Self-Transcendence (α_INDIA_ = .77; α_US_ = .75), six indicators measured the 4 values covering Openness to Change (α_INDIA_ = .71; α_US_ = .76), four indicators measured the 4 values covering Self-Enhancement (α_INDIA_ = .79; α_US_ = .81), and six indicators measured the 6 values covering Conservation (α_INDIA_ = .69; α_US_ = .77). Model fit of the unconstrained model was unacceptable: *χ^2^*(366) = 1436.84, *p* < .001; CFI = .79; RMSA = .08, 90% CI [.08; .09], *p* < .001; SRMR = .08.

Modification indices and residual inspection were used to remove offending indicators and reach configural invariance. Humility and religious tradition, for instance, showed low factor loadings and high residuals in India, these two indicators being more closely associated with self-enhancement than conformity. Similarly, gratification, prestige, and personal security didn’t fit the expected value continuum in India, and were therefore removed to meet configural invariance in the two countries. Although *χ^2^* statistics should be interpreted carefully because based on a different set of observed indicators, this new value configuration showed far better fit to the data than the previous one, Δ *χ^2^*(170) = – 824.80, *p* < .001, Δ BIC = – 15867, Δ CFI = .09. To capture more nuanced value configurations within each higher-ordered factor (i.e., benevolence, stimulation, hedonism, self-direction, achievement, power on resources, conformity), error terms were allowed to vary when both empirically and theoretically justified. This final measurement model revealed good fit to the data and was therefore retained, *χ^2^*(185) = 407.37, *p* < .001; CFI = .94; RMSA = .05, 90% CI [.04; .06], *p* = .44; SRMR = .05.

As soon as configural invariance was met, increasing levels of measurement and structural invariance were tested. As specified in Table S.1, metric invariance was almost fully met, Δ *χ^2^*(9) = 15.16, *p* = .09, Δ BIC = 9, Δ CFI = – .001, strong scalar invariance was partially met, Δ *χ^2^*(12) = 19.83, *p* = .07, Δ BIC = 9, Δ CFI = – .002, and strict residual invariance was almost fully met, Δ *χ^2^*(25) = 37.08, *p* = .06, Δ BIC = – 8, Δ CFI = – .003 (exact wording and full information of measurement invariance for each indicator is provided in Table S.1).

**Multi-dimensional COVID-19 Threats.** We assessed 10 threat subdimensions related to the COVID-19 outbreak using 30 items previously validated by BLINDED. For each statement, respondent indicated to what extent you feel worried or concerned about the pandemic situation on a scale ranging from 1 (*Not at all concerned*) to 7 (*Extremely concerned*). Three indicators for each subdimension measured the following threats: personal health (α_INDIA_ = .60; α_US_ = .80), existential (α_INDIA_ = .85; α_US_ = .89), relational (α_INDIA_ = .70; α_US_ = .89), lifestyle (α_INDIA_ = .84; α_US_ = .89), basic supply (α_INDIA_ = .85; α_US_ = .93), financial (α_INDIA_ = .87; α_US_ = .97), healthcare system (α_INDIA_ = .83; α_US_ = .96), social fabric (α_INDIA_ = .83; α_US_ = .92), political (α_INDIA_ = .74; α_US_ = .71), and vulnerable groups (α_INDIA_ = .69; α_US_ = .90). Model fit of the unconstrained model was excellent: *χ^2^*(720) = 1120.06, *p* < .001; CFI = .97; RMSA = .04, 90% CI [.03; .04], *p* > .99; SRMR = .04.

Modification indices and residual inspection did not show any offending indicator or residual variance between error terms. Thus, increasing levels of measurement and structural invariance were tested. As specified in Table S.3, metric invariance was almost fully met, Δ *χ^2^*(18) = 23.70, *p* = .17, Δ BIC = 18, Δ CFI = .000, strong scalar invariance was partially met, Δ *χ^2^*(31) = 42.62, *p* = .08, Δ BIC = 31, Δ CFI = .000, and strict residual invariance was partially met, Δ *χ^2^*(37) = 49.85, *p* = .08, Δ BIC = 37, Δ CFI = – .001 (exact wording and full information of measurement invariance for each indicator is provided in Table S.2).

**Bonding and bridging prosocial helping nintentions.** Bonding and prosocial types of prosociality were measured using items adapted from Politi and colleagues (Politi et al., 2020). Three indicators measured prosociality directed towards people physically and psychologically close to participants (i.e., bonding prosocial helping intentions), for example, “I am willing to do grocery shopping for those people in my neighborhood who are in need” (α_INDIA_ = .83; α_US_ = .91). Three indicators measured prosociality directed towards people physically and psychologically distant to participants, (i.e., bridging prosocial helping intentions), for example, “I am willing to sign petition to ask for international solidarity towards other countries that are having hard times in dealing with the corona crisis” (α_INDIA_ = .71; α_US_ = .83). Model fit was excellent: *χ^2^*(16) = 35.84, *p* = .003; CFI = .99; RMSA = .05, 90% CI [.03; .07], *p* = .43; SRMR = .03.

Modification indices and residual inspection did not show any offending indicator or residual variance between error terms. Thus, increasing levels of measurement invariance were tested. As specified in Table S.4, metric invariance was fully met, Δ *χ^2^*(4) = 6.70, *p* = .15, Δ BIC = 04, Δ CFI = –.001, strong scalar invariance was partially met, Δ *χ^2^*(6) = 11.04, *p* = .09, Δ BIC = 06, Δ CFI = –.053, and strict residual invariance was partially met, Δ *χ^2^*(10) = 16.49, *p* = .09, Δ BIC = 10, Δ CFI = – .004 (exact wording and full information of measurement invariance for each indicator is provided in Table S.3).

**Structural Invariance**

To test discriminant validity and estimate covariations between basic individual values, multidimensional COVID-19 threat, and bonding and bridging prosocial helping intentions, the three scales were put together in a unique multi-group CFA. Model fit was excellent: *χ^2^*(2427) = 3512.89, *p* < .001; CFI = .95; RMSA = .03, 90% CI [.03; .04], *p* > .99; SRMR = .035, meaning that the three scales represented distinct although correlated latent factors. As better specified in Table S.2, latent variable variance invariance, Δ *χ^2^*(2) = 4.06, *p* = .13, Δ BIC = –10, Δ CFI = – .001, as well as covariance invariance, Δ *χ^2^*(77) = 101.34, *p* = .03, Δ BIC = –412, Δ CFI = – .003, were partially met (full information of structural invariance for each latent factor is provided in Table S.4).

**References**

Beaujean, A. (2014). *Latent variable modeling using R*. Routledge.

Chou, C.-P., Bentler, P. M., & Satorra, A. (1991). Scaled test statistics and robust standard errors for non-normal data in covariance structure analysis: A Monte Carlo study. *British Journal of Mathematical and Statistical Psychology*, *44*, 347–357. https://doi.org/10.1111/j.2044-8317.1991.tb00966.x

Hu, L., & Bentler, P. M. (1999). Cutoff criteria for fit indexes in covariance structure analysis: Conventional criteria versus new alternatives. *Structural Equation Modeling: A Multidisciplinary Journal*, *6*, 1–55. https://doi.org/10.1080/10705519909540118

Politi, E., Van Assche, J., Caprara, G. V., & Phalet, K. (2021). No man is an island: Psychological underpinnings of prosociality in the midst of the COVID-19 outbreak. *Personality and Individual Differences*. https://doi.org/10.1016/j.paid.2020.110534

Rosseel, Y. (2012). Lavaan: An R package for structural equation. *Journal of Statistical Software*, *48*, 1–36. https://doi.org/10.18637/jss.v048.i02

Schwartz, S. H., Cieciuch, J., Vecchione, M., Davidov, E., Fischer, R., Beierlein, C., Ramos, A., Verkasalo, M., Lönnqvist, J. E., Demirutku, K., Dirilen-Gumus, O., & Konty, M. (2012). Refining the theory of basic individual values. *Journal of Personality and Social Psychology*, *103*, 663–688. https://doi.org/10.1037/a0029393

Vandenberg, R. J. (2002). Toward a further understanding of and improvement in measurement invariance methods and procedures. *Organizational Research Methods*, *5*, 139–158. https://doi.org/10.1177/1094428102005002001

**Table S.1:** Wording, latent factors, and measurement invariance of basic individual values

| Indicator | Latent factor | Wording | *λ* | *τ* | *θ* |
| --- | --- | --- | --- | --- | --- |
| BEN1 | Self-transcendence | It's very important to him to help the people around him. He wants to care for other people. | **✓** | **✓** | **✓** |
| BEN2 | Self-transcendence | It is important to him to be loyal to his friends. He wants to devote himself to people close to him | **✓** | **✓** | **✓** |
| UNI3 | Self-transcendence | He thinks it is important that every person in the world be treated equally. He wants justice for everybody, even for people he doesn’t know. | **✓** | ✖ | **✓** |
| UNI4 | Self-transcendence | It is important to him to listen to people who are different from him. Even when he disagrees with them, he still wants to understand them. | ✖ | ✖ | **✓** |
| UNI5 | Self-transcendence | He strongly believes that people should care for nature. Looking after the environment is important to him | **✓** | ✖ | ✖ |
| SDI6 | Openness to change | Thinking up new ideas and being creative is important to him. He likes to do things in his own original way | ✖ | ✖ | ✖ |
| SDI7 | Openness to change | It is important to him to make his own decisions about what he does. He likes to be free to plan and to choose his activities for himself. | ✖ | ✖ | **✓** |
| STI8 | Openness to change | He likes surprises and is always looking for new things to do. He thinks it is important to do lots of different things in life. | ✖ | ✖ | **✓** |
| STI9 | Openness to change | He looks for adventures and likes to take risks. He wants to have an exciting life. | **✓** | ✖ | ✖ |
| HED10 |  | Having a good time is important to him. He likes to “spoil” himself. | No configural invariance | | |
| HED11 | Openness to change | He seeks every chance he can to have fun. It is important to him to do things that give him pleasure. | **✓** | ✖ | **✓** |
| ACH12 |  | It is very important to him to show his abilities. He wants people to admire what he does. | No configural invariance | | |
| ACH13 | Self-enhancement | Being very successful is important to him. He likes to impress other people. | **✓** | ✖ | **✓** |
| POW14 | Self-enhancement | It is important to him to be rich. He wants to have a lot of money and expensive things. | **✓** | **✓** | **✓** |
| POW15 | Self-enhancement | It is important to him to be in charge and tell others what to do. He wants people to do what he says | **✓** | ✖ | **✓** |
| SEC16 |  | It is important to him to live in secure surroundings. He avoids anything that might endanger his safety | No configural invariance | | |
| SEC17 | Conservation | It is very important to him that his country be safe from threats from within and without. He is concerned that social order be protected. | **✓** | ✖ | **✓** |
| TRA18 | Conservation | Religious belief is important to him. He tries hard to do what his religion requires. | **✓** | ✖ | **✓** |
| CON19 | Conservation | He believes that people should do what they're told. He thinks people should follow rules at all times, even when no-one is watching. | **✓** | ✖ | **✓** |
| CON20 |  | It is important to him to always behave properly. He wants to avoid doing anything people would say is wrong. | No configural invariance | | |
| HUM21 |  | He thinks it's important not to ask for more than what you have. He believes that people should be satisfied with what they have. | No configural invariance | | |

*Note:* Indicator names, latent factors, and wordings are reported on the left-hand side. Group invariance of factor loadings (λ - lambda), intercepts (τ - tau), residual variance (θ – thera) are reported on the right-hand side. ✓ Invariance met ; ✖Invariance not met. Model fit: *χ^2^*(209) = 444.17, *p* < .001; CFI = .93; RMSA = .05, 90% CI [.04; .05], *p* = .62; SRMR = .06.

**Table S.2:** Wording, latent factors, and measurement invariance of the COVID-19 multifaced threat scale

| Indicator | Latent factor | Wording | *λ* | *τ* | *θ* |
| --- | --- | --- | --- | --- | --- |
| CovTh1 | Political | The government's response to the coronavirus is being used for political gains. | ✖ | ✖ | ✖ |
| CovTh2 | Political | There is so much misinformation being spread for political purposes. | **✓** | ✖ | ✖ |
| CovTh3 | Political | The Government is unable to deal with the COVID-19 pandemic. | **✓** | ✖ | **✓** |
| CovTh4 | Healthcare system | Local intensive care facilities cannot handle the impact of the virus. | **✓** | ✖ | ✖ |
| CovTh5 | Healthcare system | The hospitals are struggling to cope with the demands they are under | **✓** | **✓** | ✖ |
| CovTh6 | Healthcare system | Medical staff are unable to keep up with what is needed of them. | **✓** | **✓** | ✖ |
| CovTh7 | Vulnerable groups | The virus will impact humanitarian work in regions of conflict (e.g. Syria, Yemen). | **✓** | **✓** | ✖ |
| CovTh8 | Vulnerable groups | COVID-19 is spreading through refugee camps. | ✖ | ✖ | ✖ |
| CovTh9 | Vulnerable groups | Homeless people are not able to protect themselves | **✓** | **✓** | ✖ |
| CovTh10 | Social fabric | There are too many irresponsible people who do not respect social distancing | **✓** | ✖ | ✖ |
| CovTh11 | Social fabric | People don't seem to care that they might be infected and pass on COVID-19 | **✓** | ✖ | ✖ |
| CovTh12 | Social fabric | People simply don't respect governments orders that are designed to contain COVID-19 | **✓** | ✖ | ✖ |
| CovTh13 | Financial | My financial situation is less stable | **✓** | **✓** | ✖ |
| CovTh14 | Financial | I might run out of money | **✓** | ✖ | ✖ |
| CovTh15 | Financial | I might not be able to pay my bills | **✓** | **✓** | ✖ |
| CovTh16 | Basic supplies | There's a shortage of sanitizers and hygiene products | **✓** | **✓** | ✖ |
| CovTh17 | Basic supplies | There are shortages of essential goods (e.g., toilet paper, water). | **✓** | **✓** | ✖ |
| CovTh18 | Basic supplies | There could be food shortages in supermarkets | **✓** | **✓** | ✖ |
| CovTh19 | Lifestyle | I don't know when my next vacation will be | **✓** | ✖ | **✓** |
| CovTh20 | Lifestyle | I do not know when I will be able to travel again | **✓** | ✖ | ✖ |
| CovTh21 | Lifestyle | I can no longer visit places outside the area where I live (e.g., seaside, mountains, countryside). | **✓** | ✖ | **✓** |
| CovTh22 | Relational | I have had to become less social | **✓** | ✖ | ✖ |
| Table continues from the above page | | | | | |
| CovTh23 | Relational | I miss my friends | **✓** | ✖ | ✖ |
| CovTh24 | Relational | The lack of social contact is noticeable | **✓** | ✖ | ✖ |
| CovTh25 | Existential | I have a sense of uselessness since the pandemic started | **✓** | **✓** | **✓** |
| CovTh26 | Existential | My life has less meaning these days | **✓** | ✖ | ✖ |
| CovTh27 | Existential | I feel trapped with no way to escape | **✓** | ✖ | **✓** |
| CovTh28 | Personal health | I might catch COVID-19 | **✓** | **✓** | **✓** |
| CovTh29 | Personal health | I am very paranoid about germs these days | **✓** | **✓** | ✖ |
| CovTh30 | Personal health | I am following all the advice to avoid getting sick | **✓** | **✓** | ✖ |

*Note:* Indicator names, latent factors, and wordings are reported on the left-hand side. Group invariance of factor loadings (λ - lambda), intercepts (τ - tau), residual variance (θ – thera) are reported on the right-hand side. ✓ Invariance met; ✖Invariance not met.

**Table S.3:** Wording, latent factors, and measurement invariance of bonding and bridging prosocial helping intentions

| Indicator | Latent factor | Wording | *λ* | *τ* | *θ* |
| --- | --- | --- | --- | --- | --- |
| Comm1 | Bonding prosociality | Do grocery shopping for those people in my neighborhood who are in need. | **✓** | ✖ | **✓** |
| Comm2 | Bonding prosociality | Assist people in my hometown with disabilities in their daily activities at home | **✓** | ✖ | **✓** |
| Comm3 | Bonding prosociality | Bring medicines and medical supplies to the elderly in my community | **✓** | ✖ | **✓** |
| Global1 | Bridging prosociality | Sign petition to ask for international solidarity towards other countries that are having hard times in dealing with the corona crisis. | **✓** | **✓** | ✖ |
| Global2 | Bridging prosociality | Volunteer for the Center for Global Development currently organizing a coordinated response to the corona pandemic in poor countries. | **✓** | ✖ | **✓** |
| Global3 | Bridging prosociality | Donate money for Non-Governmental Organizations who are providing medical supplies to Latino refugees living in camps in Mexico and Guatemala. | **✓** | **✓** | ✖ |

*Note:* Indicator names, latent factors, and wordings are reported on the left-hand side. Group invariance of factor loadings (λ - lambda), intercepts (τ - tau), residual variance (θ – thera) are reported on the right-hand side. ✓ Invariance met; ✖Invariance not met.

**Table S.4:** Structural invariance of the multidimensional COVID-19 threat scale

|  | ST | OC | SE | CO | PL | HS | VG | SF | FN | BS | LF | RL | EX | PH | BoP | BrP |
| --- | --- | --- | --- | --- | --- | --- | --- | --- | --- | --- | --- | --- | --- | --- | --- | --- |
| *Basic individual values* |  |  |  |  |  |  |  |  |  |  |  |  |  |  |  |  |
| Self-transcendence (ST) | ✖ |  |  |  |  |  |  |  |  |  |  |  |  |  |  |  |
| Openness to change (OC) | **✓** | ✖ |  |  |  |  |  |  |  |  |  |  |  |  |  |  |
| Self-enhancement (SE) | **✓** | ✖ | **✓** |  |  |  |  |  |  |  |  |  |  |  |  |  |
| Conservation (CN) | ✖ | **✓** | ✖ | ✖ |  |  |  |  |  |  |  |  |  |  |  |  |
| *COVID-19 threats* |  |  |  |  |  |  |  |  |  |  |  |  |  |  |  |  |
| Political (PL) | **✓** | **✓** | **✓** | ✖ | ✖ |  |  |  |  |  |  |  |  |  |  |  |
| Healthcare system (HS) | **✓** | **✓** | **✓** | ✖ | **✓** | ✖ |  |  |  |  |  |  |  |  |  |  |
| Vulnerable groups (VG) | **✓** | **✓** | **✓** | ✖ | **✓** | ✖ | ✖ |  |  |  |  |  |  |  |  |  |
| Social fabric (SF) | **✓** | **✓** | **✓** | ✖ | **✓** | ✖ | ✖ | **✓** |  |  |  |  |  |  |  |  |
| Financial (FN) | **✓** | ✖ | **✓** | **✓** | ✖ | ✖ | **✓** | ✖ | ✖ |  |  |  |  |  |  |  |
| Basic supplies (BS) | **✓** | ✖ | **✓** | **✓** | **✓** | ✖ | ✖ | **✓** | ✖ | ✖ |  |  |  |  |  |  |
| Lifestyle (LF) | **✓** | ✖ | **✓** | ✖ | **✓** | **✓** | ✖ | ✖ | **✓** | **✓** | ✖ |  |  |  |  |  |
| Relational (RL) | **✓** | ✖ | **✓** | **✓** | **✓** | **✓** | **✓** | **✓** | **✓** | **✓** | ✖ | ✖ |  |  |  |  |
| Existential (EX) | **✓** | **✓** | ✖ | **✓** | ✖ | **✓** | **✓** | **✓** | **✓** | ✖ | **✓** | ✖ | ✖ |  |  |  |
| Personal health (PH) | **✓** | **✓** | **✓** | ✖ | **✓** | ✖ | ✖ | ✖ | **✓** | ✖ | **✓** | ✖ | **✓** | ✖ |  |  |
| *Prosocial intentions* |  |  |  |  |  |  |  |  |  |  |  |  |  |  |  |  |
| Bonding prosociality (BoP) | **✓** | **✓** | ✖ | **✓** | ✖ | ✖ | **✓** | ✖ | **✓** | **✓** | **✓** | **✓** | **✓** | ✖ | ✖ |  |
| Bridging prosociality (BrP) | **✓** | **✓** | **✓** | ✖ | **✓** | **✓** | ✖ | **✓** | **✓** | **✓** | **✓** | **✓** | **✓** | **✓** | ✖ | ✖ |

*Note:* Latent variable variance invariance (θ – thera) is reported along the table diagonal. Latent variable covariance invariance (φ – phi) is reported below the diagonal. ✓ Invariance met; ✖Invariance not met.

**Table S.5:** Variance/covariance matrix latent factors

|  | ST | OC | SE | CO | PL | HS | VG | SF | FN | BS | LF | RL | EX | PH | BoP | BrP |
| --- | --- | --- | --- | --- | --- | --- | --- | --- | --- | --- | --- | --- | --- | --- | --- | --- |
| Self-transcendence (ST) | 0.53  *0.42* |  |  |  |  |  |  |  |  |  |  |  |  |  |  |  |
| Openness to change (OC) | 0.34^***^ | 0.39  *1.28* |  |  |  |  |  |  |  |  |  |  |  |  |  |  |
| Self-enhancement (SE) | – 0.05 | 0.15^**^  *0.53^***^* | 1.14 |  |  |  |  |  |  |  |  |  |  |  |  |  |
| Conservation  (CN) | 0.36^***^  *0.16^***^* | 0.30^***^ | 0.24^***^  *0.63^***^* | 0.58  *1.25* |  |  |  |  |  |  |  |  |  |  |  |  |
| Political  (PL) | 0.11^***^ | 0.05 | – 0.02 | 0.09  *–0.17^**^* | 1.44  *0.55* |  |  |  |  |  |  |  |  |  |  |  |
| Healthcare system (HS) | 0.29^***^ | 0.12^**^ | – 0.05 | 0.40*^***^*  *–0.28^**^* | 0.96^***^ | 1.50  *2.59* |  |  |  |  |  |  |  |  |  |  |
| Vulnerable groups (VG) | 0.40^***^ | 0.12^**^ | –0.07 | 0.34*^***^*  *–0.29^***^* | 0.78^***^ | 0.87^***^  *1.63^***^* | 1.45  *2.42* |  |  |  |  |  |  |  |  |  |
| Social fabric  (SF) | 0.29^***^ | 0.12^**^ | – 0.08 | *0.36^***^*  *–0.22^*^* | 0.79^***^ | 1.10^***^  *1.52^***^* | 0.97^***^  *1.14^***^* | 1.82 |  |  |  |  |  |  |  |  |
| Financial  (FN) | 0.12^*^ | 0.001  *0.27^*^* | 0.41^***^ | 0.29^***^ | 0.47^***^  *0.24^**^* | 0.89^***^  *0.50^**^* | 0.66^***^ | 0.60^***^  *0.19* | 2.70  *4.96* |  |  |  |  |  |  |  |
| Basic supplies  (BS) | 0.21^***^ | 0.09  *0.39^***^* | 0.26^**^ | 0.34^***^ | 0.43^***^ | 0.78^***^  *1.20^***^* | 0.82^***^  *0.98^***^* | 0.59^***^ | 1.33^***^  *1.91^***^* | 2.61  *3.22* |  |  |  |  |  |  |
| Lifestyle  (LF) | 0.11^*^ | 0.09  *0.56^***^* | 0.61^***^ | 0.16^†^  *0.51^***^* | 0.24^***^ | 0.24^*^ | 0.66^***^  *0.30^*^* | 0.48^***^  *0.20* | 0.80^***^ | 0.83^***^ | 2.97  *3.34* |  |  |  |  |  |
| Relational  (RL) | 0.23^***^ | 0.13^**^  *0.44^***^* | 0.38^***^ | 0.30^***^ | 0.35^***^ | 0.51^***^ | 0.52^***^ | 0.44^***^ | 0.65^***^ | 0.72^***^ | 1.17^***^  *1.79^***^* | 1.42  *2.82* |  |  |  |  |
| Existential  (EX) | –0.05 | 0.03 | 0.82^***^  *0.54^***^* | 0.12 | 0.57^***^  *0.31^***^* | 0.59^***^ | 0.55^***^ | 0.22^*^ | 1.46^***^ | 1.30^***^  *0.86^***^* | 1.41^***^ | 1.07^***^  *1.94^***^* | 2.91  *3.82* |  |  |  |
| Personal health (PH) | 0.20^***^ | 0.06 | 0.22^**^ | 0.46^***^ | 0.77^***^ | 1.00^***^  *1.51^***^* | 0.88^***^  1.23^***^ | 1.12^***^  *1.30^***^* | 0.99^***^ | 0.95^***^  *1.48^***^* | 1.11^***^  *1.87^***^* | 0.79^***^  *1.01^***^* | 1.19^***^ | 1.52  *2.47* |  |  |
| Bonding prosociality (BoP) | 0.45^***^ | 0.31^***^ | – 0.07  *0.03* | 0.36^***^  *0.20^*^* | 0.33^***^  *–0.04* | 0.51^***^  *–0.13* | 0.47^***^ | 0.53^***^  *0.02* | 0.14 | 0.21^*^ | 0.05 | 0.18^*^ | –0.11 | 0.38^***^  *0.003* | 1.56  *3.07* |  |
| Bridging prosociality (BrP) | 0.44^***^ | 0.31^***^ | 0.14^*^ | 0.38^***^  0.04 | 0.27^***^ | 0.49^***^ | 0.62*^***^*  *1.03^***^* | 0.50^***^ | 0.39^**^ | 0.51^***^ | 0.20 | 0.40^***^ | 0.27^*^ | 0.46^***^ | 1.24^***^  *2.03^***^* | 1.67  *2.70* |

*Note:* Latent variable variance (θ – thera) is reported along the table diagonal. Latent variable covariance (φ – phi) is reported below the diagonal. When country invariance was not met, US estimates are reported in italic. ^†^ *p <* .10, ^*^ *p* < .05*, ^**^ p* <.01, *^***^ p* <.001.
